# “I could not find the strength to resist the pressure of the medical staff, to refuse to give commercial milk formula”: a qualitative study on effects of the war on Ukrainian women’s infant feeding

**DOI:** 10.3389/fnut.2024.1225940

**Published:** 2024-05-17

**Authors:** Alessandro Iellamo, Christina Misa Wong, Oleg Bilukha, Julie P. Smith, Mija Ververs, Karleen Gribble, Bartłomiej Walczak, Aleksandra Wesolowska, Sura Al Samman, Michael O’Brien, Annette N. Brown, Tobias Stillman, Blythe Thomas

**Affiliations:** ^1^Nutrition Unit, FHI 360, Washington, DC, United States; ^2^Global Health and Population Research, FHI 360, Durham, NC, United States; ^3^Global Public Health Emergency Branch, Global Health Center, Centers for Disease Control and Prevention, Atlanta, GA, United States; ^4^National Centre for Epidemiology and Population Health, The Australian National University, Canberra, NSW, Australia; ^5^School of Nursing and Midwifery, Western Sydney University, Parramatta, NSW, Australia; ^6^Institute of Applied Social Sciences, University of Warsaw, Warsaw, Poland; ^7^Laboratory of Human Milk and Lactation Research, Department of Medical Biology, Faculty of Health Sciences, Medical University of Warsaw, Warsaw, Poland; ^8^Human Milk Bank Foundation, Warsaw, Poland; ^9^Jordan Community Health and Nutrition Behavior Change Project, FHI 360 WAMERO, Amman, Jordan; ^10^Crisis Response Unit, FHI 360, Washington, DC, United States; ^11^Strategy and Innovation with Evidence Unit, FHI 360, Washington, DC, United States; ^12^1000 Days Initiative, FHI Solutions, Washington, DC, United States

**Keywords:** infant feeding in emergencies, IYCF-E, breastfeeding, IYCF, breastfeeding in emergencies, infant formula feeding, infant feeding, emergency nutrition

## Abstract

**Introduction:**

During emergencies, breastfeeding protects infants by providing essential nutrients, food security, comfort, and protection and is a priority lifesaving intervention. On February 24, 2022, the war in Ukraine escalated, creating a humanitarian catastrophe. The war has resulted in death, injuries, and mass internal displacement of over 5 million people. A further 8.2 million people have taken refuge in neighboring countries, including Poland. Among those impacted are infants and young children and their mothers. We conducted a study to explore the infant feeding challenges and needs of Ukrainian women affected by the war.

**Methods:**

We conducted a qualitative descriptive study involving in-depth interviews (IDIs) with 75 war-affected Ukrainian mothers who had at least one infant aged less than 12 months at the time of the interview. Eligible mothers were either (1) living as Ukrainian refugees in Poland, having crossed the border from Ukraine on or after February 24, 2022, when the war started (*n* = 30) or (2) living in Ukraine as internally displaced persons or as residents in the community (*n* = 45). All interviews were audio-recorded (either transcribed or had responses summarized as expanded notes) and analyzed using qualitative thematic analysis using a two-step rapid analysis process.

**Results:**

Participants in Ukraine who wanted to initiate breastfeeding right after birth faced opposition from healthcare workers at maternity hospitals. Ukrainian refugees who gave birth in Poland faced language barriers when seeking breastfeeding support. Half of the participants in Ukraine received commercial milk formula (CMF) donations even if they said they did not need them. Most respondents stated that breastfeeding information and support were urgently needed.

**Conclusion:**

Our data suggests that healthcare workers in Ukrainian maternity hospitals require additional training and motivation on delivering breastfeeding support. In addition, lactation consultants in maternity ward are needed in Ukraine, and interpretation support is needed for refugees to overcome language barriers. There is a need to control the indiscriminate donations of commercial milk formula and to ensure that complementary foods and commercial milk formula are available to those that need it. This study confirms the need for actions to ensure infant and young child feeding (IYCF) support is provided during emergencies.

## 1 Introduction

The deaths of more than 820,000 children younger than 5 years can be prevented each year by improving breastfeeding practices (1). Breastfeeding is the most cost-effective intervention to improve child survival, reduce morbidity, and ensure proper growth and wellbeing. The importance of initiation of breastfeeding within 1 hour of birth, exclusive breastfeeding for the first 6 months of life, and continued breastfeeding for at least 2 years after birth has been well documented (1). In all contexts, breastmilk also lowers children’s risk of non-communicable diseases later in life (2). Breastfed children are found to perform better on intelligence tests, have higher school attendance, and have higher incomes in adult life (1, 3). Longer duration of breastfeeding also contributes to the health and wellbeing of the mothers, including reducing the risk of ovarian and breast cancers and helping to space pregnancies (1). The economic consequences of cognitive losses and conservative estimates of reduced treatment costs of childhood diseases suggest that the economic benefits for all countries of promoting breastfeeding are likely to be substantial, and the losses associated with premature cessation of breastfeeding amount to $302 billion annually, or 0.49 percent of world gross national income (GNI) (4).

During emergencies, breastfeeding is a shield that protects infants by providing food security, comfort, warmth, and protection and is a priority lifesaving intervention (1, 5). However, the emergency response related to infant and young child feeding (IYCF) is often less than adequate. In 2017, fewer than 30 percent of World Health Organization (WHO) member states had relevant IYCF in emergencies (IYCF-E) recommendations (6). Yet, as long ago as in 2001, the Infant Feeding in Emergencies (IFE) Core Group, of which UNICEF and WHO are members, issued the Operational Guidance for Infant and Young Child Feeding in Emergencies (OG-IFE) (7). In 2004, the WHO issued guiding principles for feeding infants and young children during emergencies (8). In 2010, the World Health Assembly (WHA 63.23) (9) endorsed the OG-IFE. In 2017, the updated OG-IFE was released (7). In 2018, the World Health Assembly (WHA 71.9) urged member states to “*take all necessary measures to ensure evidence-based and appropriate infant and young child feeding during emergencies*” (10). Also, in 2018, the new Sphere Handbook was issued, which included a specific set of IYCF-E recommendations (11).

More than 20 years since the release of the first IYCF-E Operational Guidance, humanitarian responses have shown some progress in protecting the health of infants and young children through appropriate feeding support. For example, during the Haiti earthquake in 2010, baby tents were introduced to provide a safe place for mothers to breastfeed and for non-breastfed infants (12). Following the 2011 Christchurch, New Zealand earthquake, an emergency day-stay breastfeeding service assisted evacuee women (13). However, many emergency responses have failed to invest in the protection of infants and young children through appropriate IYCF-E support. For example, the women and children affected by the flood that hit the state of Kelantan, Malaysia, in December 2014 were concerned about the lack of privacy for mothers to breastfeed their babies comfortably and the uncontrolled donations of commercial infant formula, teats, and feeding bottles (14).

In Ukraine, the 2014 hostilities in the eastern oblasts of Donetsk and Luhansk affected millions of people, including women and their infants. A study reviewed the IYCF practices of the affected populations and found that, in general, a majority of women had breastfed their children (93.3%). However, the same study found a very low prevalence of critical IYCF-recommended practices, including a low prevalence of exclusive breastfeeding and a very high prevalence of commercial milk formula usage (15). These findings were comparable with the IYCF situation pre-conflict, where 96.7% of children were ever breastfed, and 21.3% of infants less than 6 months of age were exclusively breastfed (15).

After the 2014 hostilities, efforts were supported by the Government of Ukraine to improve the breastfeeding situation in the country, and the Baby Friendly Hospital Initiative, with the certification covering 30% of the mother and child health facilities, was invested in. In 2021, the President of Ukraine approved a law to comply with the European Regulations that aims to implement the recommendations and standards of the International Code of Marketing of Breastmilk Substitutes (16).

In a recent international conference, the Ministry of Health presented that more than 92% of health facilities in Ukraine were certified Baby-Friendly by the Ministry of Health. Exclusive breastfeeding during the first 6 months was recorded to be between 56 and 76%, suggesting some improvements compared with the period prior to the 2014 conflict (16).

Unfortunately, on February 24, 2022, a new war in Ukraine escalated, creating a humanitarian catastrophe. As of early 2023, there were 17.6 million people in need of humanitarian assistance, including 4.1 million children (17). UN Women also estimates that around 265,000 Ukrainian women were pregnant when the war broke out (18, 19).

We conducted a study to explore the infant feeding challenges and needs of Ukrainian women affected by the war. We included Ukrainian women who remained in Ukraine, and also Ukrainian women who had crossed the border into Poland as refugees after February 24, 2022 when the war started. We did this as we wanted to explore differences in the emergency responses in both countries and how that impacted women’s behaviors as they relate to IYCF. The results of our study will inform forums and discussions around the need for more actions and resources to ensure women and their infants are protected and supported to practice safe and appropriate feeding during emergency responses.

## 2 Materials and methods

### 2.1 Study design and participants

In this qualitative descriptive study, we conducted in-depth interviews (IDI) with Ukrainian mothers of infants less than 12 months old. We purposively sampled participants to include mothers who had at least one infant aged less than 12 months at the time of the interview and who were either (1) a refugee living in Poland who had crossed the border from Ukraine on or after February 24, 2022, when the war started (“refugee”) or (2) living in Ukraine as an internally displaced person in a shelter or as a resident in the community (“participant in Ukraine”). All participants had to be 18 years or older. Local study teams in each country recruited mothers through local networks such as non-governmental organizations, health centers, and hospitals or through Facebook, a social networking site.

There was a total of nine female interviewers (two in Poland and seven in Ukraine). All interviewers had previous interviewing experience and participated in study-specific training, including the review of the IDI guides. The training was conducted virtually by the FHI 360 lead investigator (AI) for the interviewers in Ukraine, while experienced researchers from the Medical University of Warsaw (AW) and the University of Warsaw (BW) conducted the training for the interviewers in Poland. In both cases, the training lasted half a day. Interviewers were not previously acquainted with the study participants.

### 2.2 Data collection

We conducted a total of 75 IDIs (30 in Poland and 45 in Ukraine) between September 5 and 26, 2022. We determined this sample size based on previous research that demonstrated that data saturation occurs by 12 interviews for relatively homogenous samples (which for our study was for each country) (20). Given uncertainty regarding the homo- or heterogeneity of our samples, we interviewed more participants than this to ensure data saturation and inclusion of participants from diverse regions in north and central Poland and eastern and western zones in Ukraine. Prior to initiating data collection, interviewers introduced themselves and the study objectives. Participants were then screened for eligibility and if they were eligible, they were then brought through the informed consent form. Participants provided oral informed consent to participate in an audio-recorded IDI. In Poland, IDIs were conducted by phone (*n* = 19) and through web-based platforms (such as Zoom) (*n* = 11), while IDIs in Ukraine were conducted through web-based platforms (*n* = 30) and in-person (*n* = 15). These in-person IDIs in Ukraine were conducted at shelters and social service centers for women and children. One-on-one interviews were conducted in Ukrainian or Russian language, depending on the participants’ preference. These interviews lasted between 15 and 77 min.

A semi-structured interview guide was used during the interview. The interview topic domains can be found in Table 1. The interview guide was not pilot-tested but was reviewed by two study team members, both Ukrainian mothers, one of whom is also a refugee in Poland.

**TABLE 1:** Topic domains discussed in the interviews with mothers.

• Information on children • Number of children • Youngest child’s age • Youngest child’s birth country • Experience with the pregnancy of youngest child and that child’s health information • Feeding practices of the youngest child • Feeding plans before birth and whether those plans changed after the child was born • Feeding practices of youngest child right after birth • Feeding practices of youngest child before war started • Influence of war on feeding practices of youngest child • Changes of plan to youngest child’s diet • Support • Support in feeding youngest child in Ukraine and Poland • Suggestions for improvement to support infant feeding and the wellbeing of families during conflict

The interviewers collected socio-demographic data at the end of each IDI. All IDIs were audio-recorded, and study team members transcribed the audio recordings or wrote expanded notes immediately after each IDI in the language that the interviews were conducted. All transcripts and expanded notes were then translated into English by professional Ukrainian translators in Poland and Ukraine. Interviewers reviewed the translated transcripts and expanded notes to ensure translation accuracy. We did not return the transcripts to participants for comments or corrections.

### 2.3 Data analysis

Qualitative thematic analysis was conducted using a two-step rapid analysis process (21). This two-step rapid analysis approach limits in-depth analysis as compared to traditional thematic analysis, but the approach is still methodologically rigorous (22). In step one, a structured template was developed for each of the two participant types (i.e., refugees in Poland and participants in Ukraine) using Microsoft Excel. In this template, each column corresponds to a topic domain from the IDI guide (Table 1), with a row for each transcript or expanded notes. Three analysts (including AI and CMW) populated the template by reviewing the transcripts/expanded notes one by one to summarize the responses for each topic domain. This allowed responses from different parts of the transcript to be collated under a topic domain. Relevant illustrative quotes were extracted and added to the same cell as the summarized response.

In step two, the same three analysts were assigned different topic domains to review. For each topic domain, analysts reviewed the template entries for that domain and developed summary reports to reduce and interpret the data by participant type (i.e., refugees in Poland and participants in Ukraine) and by whether their youngest child was born before or after the war started in Ukraine. These summary reports described themes and sub-themes and included illustrative quotes. Socio-demographic data were analyzed descriptively by participant type.

While conducting data analysis, we made sure that data saturation had been reached and that there was no need to conduct additional interviews. The participants did not have access to the study findings for their review and feedback. We used the Consolidated Criteria for Reporting Qualitative Research (COREQ) checklist to report on the study results (23).

### 2.4 Ethics approval

Ethical approval was obtained from the Bioethics Committee at Warsaw Medical University (AKB/239/2022). This activity also received a non-research determination by FHI 360’s Office of International Research Ethics (IRBNet ID: 1952841-1).

## 3 Results

### 3.1 Socio-demographic characteristics

Of the 75 interviews conducted with Ukrainian mothers, 30 were with refugees in Poland and 45 with participants in Ukraine. Refugees in Poland were primarily located in Krakow and Wrocław, while participants in Ukraine were from oblasts covered all regions of Ukraine–West, Center, South and East.

Socio-demographic characteristics of mothers are presented in Table 2. The median age for the two participant groups was similar. Refugee mothers had a median of two children, while participants in Ukraine had a median of one child. Most mothers, both refugees and participants in Ukraine (73 and 84%, respectively), had university-level education. About half of the youngest children of refugees and participants in Ukraine were less than 6 months old, while the other half were between 6 and 11.9-months old. Among refugees, under half (43%) of their youngest children were born in Ukraine before the war, while the rest were born after the war, mostly in Poland. Of the participants in Ukraine, half (53%) gave birth to their youngest child in Ukraine before the war, while the other half were born in Ukraine after the war started.

**TABLE 2:** Socio-demographic characteristics of mothers (*n* = 75).

	Refugees (Poland) *N* = 30	Participants in Ukraine *N* = 45
Mothers’ median age (range)	33.5 years (18–40 years)	31 years (18–40 years)
Median number of children (range)	2 (1–3)	1 (1–9)
**Mother’s highest education**		
Secondary/technical	8 (26.67%)	6 (13.33%)
Higher education/university	22 (73.33%)	38 (84.44%)
**Age of youngest child at time of interview**		
<6 months old	16 (53.33%)	20 (44.44%)
6–11.9 months old	14 (46.67%)	25 (55.56%)
**When and where youngest child was born**		
Before the war (in Ukraine)	13 (43.33%)	24 (53.33%)
After the war		
In Ukraine	2 (6.67%)	21 (46.67%)
In Poland	15 (50%)	

### 3.2 Feeding practices at birth before the war started

About half of the infants born in Ukraine before the war were exclusively breastfed in the hospital after their birth, while about a quarter were given only commercial milk formula (CMF), and the remaining quarter were fed with a mix of breastmilk and CMF. Participants were asked if they received breastfeeding support right after birth from staff at the health facility where their youngest child was born. Just under a quarter of the participants who gave birth before the war said that they had received breastfeeding support. Mothers who were breastfeeding their infants said that they felt encouraged and motivated by healthcare workers who supported them, as this participant describes:

*In the ward, I was supported by the medical staff regarding breastfeeding, encouraged and praised that I was giving my baby expressed milk, because it is very good for the baby (Participant in Ukraine, the child was born in Ukraine before the war).*

### 3.3 Feeding practices at birth after the war started—For infants born in Ukraine

For infants born in Ukraine after the war started, CMF use in hospitals after birth was much more common than before the war, and we assume that it may have replaced partial breastfeeding. Just under half were exclusively breastfed, just under half were given CMF only, and a small number of others were fed a mix of breastmilk and CMF right after birth. Some mothers in Ukraine who wanted to breastfeed faced opposition from healthcare workers who urged mothers to feed with CMF, saying it would be easier because of the war, as described by two participants in Ukraine:

*Immediately after birth, they [health care workers] put my child on my chest; we were together even during the air alarm. However, when they went out to the corridor during the air raid, the nurses recommended that all mothers feed CMF to their infants because of the war. Most mothers agreed and gave CMF brought by the medical staff. However, I did not give CMF to my baby. In order not to argue with medical staff, I just agreed [to feed with CMF] and even bought a pack of CMF and a bottle and simulated feeding with the CMF. I bought CMF and the bottle at my own expense. During a routine check-up at the district pediatrician at the age of 1 month, also during the appointment, the pediatrician recommended giving CMF repeating the same mantra “war—stress—milk may disappear” (Participant in Ukraine, child was born in Ukraine after the war started).*

*When I gave birth, they [health care workers] fed CMF to the baby in the delivery room. I insisted on breastfeeding my child, but they [health care workers] latched the baby on my breast for 30 s when I asked and then took the baby away after that. My child screamed, and the doctors put pressure on me saying that my breasts were “dry” and the baby needed CMF. I tried to latch the baby by myself, but my breasts hurt a lot during latching. I asked the medical staff to help, but they said, “Don’t fool yourself; there’s nothing in your breasts; don’t torture the child” (Participant in Ukraine, child was born in Ukraine after the war started).*

Despite these healthcare workers’ opposition to breastfeeding, there were still a small number of participants in Ukraine who said that they did receive breastfeeding support right after birth from staff at the health facility. As this participant explains:

*At the hospital, I started breastfeeding. The nurse immediately came, checked the correctness of the latching. Then after a few days, mastitis began, the midwife came and pumped, it was moderately painful (Participant in Ukraine, child was born in Ukraine after the war started).*

### 3.4 Feeding practices at birth after the war started—For infants born in Poland

For infants born in Poland after the war started, about half of the infants were given only breastmilk, about a quarter were given only CMF, and the remaining quarter were fed with a mix of breastmilk and CMF. This is similar to feeding practices in Ukraine before the war started. Some mothers who gave birth in Poland felt pressure from healthcare workers to give CMF to their newborn infants. A refugee who used to be a healthcare worker in Ukraine before the war said that she had to refuse the offer of the Polish healthcare workers to give CMF to her infant:

*Yes, immediately in the delivery room, my child was put to the breast. For the first two or three days, I had colostrum, it was yellow, the infant had enough of it. True, the medical staff offered to feed the infant with CMF, but I refused. I understood that I had to wait a little and my milk would arrive (Refugee, child was born in Poland after the war started).*

A few refugees who gave birth in Poland mentioned that they did receive some breastfeeding support right after birth from healthcare workers, as this refugee explained:

*About 4 h after giving birth, a consultant came to me… She [health care worker] came with a syringe, collected the colostrum, and took it to the child in the intensive care unit. The consultant told me that I had to do the same thing every 3 h: collect the colostrum with a syringe and take it to the intensive care unit, the whole time I stayed in the hospital. Then, when I was discharged, I had to bring [to the hospital] breastmilk once a day. I did so (Refugee, child was born in Poland after the war started).*

Refugees who gave birth in Poland had additional challenges with language barriers when trying to get breastfeeding support. One refugee participant recounted her experience stating that if she had given birth in Ukraine, she would not have experienced language barriers and added stress when she encountered breastfeeding challenges:

*They [health care workers] gave me my child and said, “take it,” but I was afraid to take it, because I was afraid to harm it. I don’t know how to put him to my breast… I understood that he had to be fed, but I just couldn’t handle it physically and mentally. If I were in Ukraine, within my native walls, there would be no such stress. There would be no language barrier, there would be no stress. Even if for some reason I would also have had a caesarean section, psychologically this moment would have been easier to live (Refugee, child was born in Poland after the war started).*

Getting help in the same language was also important for a refugee who recounted her experience at the maternity hospital after she gave birth where there was a lactation counselor and a Ukrainian-speaking psychologist:

*There was a lactation specialist in the ward, who came every day and looked at the breasts. She asked if there were any problems, if I was feeding, if I had milk. There was also a Ukrainian-speaking psychologist, and you could talk in Ukrainian, it was very nice (Refugee, child was born in Poland after the war started).*

### 3.5 CMF was provided free at birth

Participants were asked if CMF provided by the health facility at birth was given for free. Most of the refugees in Poland and all participants in Ukraine who were giving CMF to their child said that they received it for free from the health facility. Mothers were asked to share the reasons given by the health facility for giving free CMF. Refugees and participants in Ukraine recalled that the main reason was that it was hospital practice to offer free CMF. Other reasons that refugees in Poland mentioned were that they were given free CMF as their child was experiencing a medical condition and could not be breastfed, the infant was born prematurely, or the mother had undergone a caesarean section delivery. Participants in Ukraine gave different reasons such as believing to have breast conditions that were contraindications to breastfeeding (e.g., inverted nipple even when this for example is not a real contraindication) or that the infant was not gaining weight or that the infant was hungry and crying.

### 3.6 Feeding practices around the time of the interview

Participants were asked what they fed their children in the 24 h before the interview. Among refugees and participants with children under 6 months old, about one-third said that they were giving only breastmilk, while another one-third said that they were giving a combination of breastmilk and CMF (Table 3). Others said their infants were fully CMF fed or were given complementary foods in the previous 24 h (early introduction of complementary feeding). Most infants between 6 and 11.9 months for both refugees and those in Ukraine were fed complementary foods together with either breastmilk or CMF.

**TABLE 3:** Types of foods participants fed their infants 24 h before the interview by age and participant group (*n* = 72).

Age/participant group	Breast milk only	Breast milk and foods	CMF only	CMF and foods	Breast milk and CMF	Foods only	Total
**Infants under 6 months old**							
Refugees	5	1	2	0	5	2	15
Participants in Ukraine	6	5	0	1	4	1	17
**Infants 6–11.9 months old**							
Refugees	0	6	0	5	2	1	14
Participants in Ukraine	1	8	0	15	1	1	26

There were no responses from 3 participants.

### 3.7 Changes to infant feeding because of the war

Participants who were pregnant before the war and gave birth after the war started were asked how they planned to feed their child and if those plans changed because of the war. Most refugees and participants in Ukraine said that they had planned before the war to exclusively breastfeed their child as they understood the importance of breastmilk. However, after the war started and after the infant was born, many refugees and participants in Ukraine said that they had changed their feeding plans. The majority of those who said that their plans changed decided to supplement breastmilk with CMF, with a small number saying that they switched completely to CMF.

A participant in Ukraine shared the feelings of guilt she was still experiencing because she was not able to breastfeed as planned due to the war:

*Yes, the war affected the feedings because I had planned to exclusively breastfeed. However, at the hospital, I fed CMF to my child. The reason for that was that I gave birth during the shelling/bombing and then became confused and could not find the strength to resist the pressure of the medical staff, to refuse to give CMF. I still feel guilty [participant starts crying] because of that, that I was not able to protect my infant from unjustified CMF feeding, and this pain has not subsided. My husband and relatives do not understand why I am being so hard on myself because of this; they do not understand what is going on. But I believe that I did not protect my child (Participant in Ukraine, child was born in Ukraine after the war started).*

We also asked all participants directly whether the war affected or influenced the way they fed their child. Most of the refugees and participants in Ukraine said that the war did affect the way they are feeding their children. Most commonly reported was the perceived reduction in breastmilk supply because of stress, worry, and anxiety due to the war:

*And then the hostilities became more active close to our place. Shelling and explosions came every 5 or 10 min. My mental health got worse. I was constantly nervous. That is why my milk supply dropped. Certainly, the war did affect the situation. My breastmilk production was not sufficient to feed the child (Participant in Ukraine, child was born after the war started in Ukraine).*

For some participants in Ukraine, the war also influenced the early introduction of complementary foods early (Table 3) as this participant in Ukraine shared:

*It [war] partially affected because I introduced solid food earlier than planned, because I was very afraid that later there would be no breastmilk due to stress and the child would not have anything to eat, so at least he would have time to get used to “adult food.” I was in fear that the breastmilk would disappear and that it would be impossible to purchase CMF anywhere (Participant in Ukraine, child was born in Ukraine before the war).*

Many said that because of the war, it was hard to find or buy food that was appropriate for their infants. Others, especially those in Ukraine, said that the war affected the availability of CMF and made it difficult to find the brand that they had been using. They also said CMF was expensive, and it was difficult to find bottles and safe water to mix the CMF with:

*Clearly, the war had an impact. You constantly watch the news; you are afraid that the milk will disappear. I am worried about whether I will be able to buy CMF. CMF is expensive. I constantly think about whether I will have enough to feed my child (Refugee, child was born in Poland after the war started).*

*I was in fear that the breastmilk would disappear and that it would be impossible to purchase CMF anywhere (Participant in Ukraine, child was born in Ukraine before the war).*

### 3.8 Infant feeding support received in Ukraine during the war

Participants were asked if they had received support with their child’s nutrition after the war started and if so, what type of support they received or accessed. Among participants in Ukraine, most said that they had received support. Half of the participants in Ukraine said that they received CMF from volunteers and charitable organizations. These donations were given even if mothers did not need the CMF as this participant in Ukraine explains:

*Humanitarian aid provided CMF. I refused to take it, but they said that I had to take it (Participant in Ukraine, child was born in Ukraine after the war started).*

Although many mothers appreciated getting CMF, one mother expressed that giving out so many cans of CMF jeopardizes breastfeeding:

*Also, I observed that a lot of CMF are given out by charitable foundations. I saw other mothers take CMF and believe that this is not good; this threatens the concept of breastfeeding (Participant in Ukraine, child was born in Ukraine before the war).*

Another participant said that her mother took the CMF that was given out by humanitarian aid in case she is not there to breastfeed:

*In humanitarian aid, they gave CMF. My mother [participant’s mother] also took them. My mother takes care of the CMF, because she has taken the position that if something happens to me, then in emergency cases, the child can be fed [with CMF] by relatives (Participant in Ukraine, child was born in Ukraine after the war started).*

Half of the participants in Ukraine also said that they received help from family members who provided support for breastfeeding, cooking, feeding, and bottle-feeding the infant. A participant in Ukraine said that her husband, mother, and mother-in-law supported her to breastfeed and helped with feeding her child with breastmilk she had previously expressed:

*I was supported by relatives—my husband, his mother, and my mother. They all advised me to breastfeed the child and gave other tips. This is how they supported me. When I had to go out to register the child, issue a birth certificate, etc…. I pumped milk, and my mother fed the child with a bottle (Participant in Ukraine, child was born in Ukraine before the war).*

One-fourth of participants said that they received breastfeeding/nutrition information from the maternity hospital where they gave birth and from pediatricians and other healthcare workers. Others said that they received financial aid from the government to buy food; food from volunteers, charitable organizations, and friends; and psychological and mental health support from friends and organizations. A participant in Ukraine said that receiving counseling from a psychologist really helped her especially with postpartum depression and building better relationships with her children:

*Now the crisis team and psychologist continue supporting my family—with advice, information, and psychological services. I have 1-h individual counseling with a psychologist twice a week. It helps me to cope with postpartum depression and build better relationships with children. I feel better after counseling with a psychologist (Participant in Ukraine, child was born in Ukraine before the war).*

A few participants in Ukraine also said that they received breastfeeding counseling from a lactation counselor and nutrition information from the internet, social media, posters, and leaflets.

### 3.9 Infant feeding support received by refugees in Poland during the war

Among refugees, two-thirds said that they received support with their child’s nutrition after the war started. Of these refugees, one-third said that they received information on breastfeeding and nutrition at the maternity hospital and from pediatricians and other healthcare workers. A refugee explained that she went for lectures on breastfeeding and breastfeeding information she had received from informational booklets:

*Yes, I have received a package for an infant where all the necessary information was some booklets about the breastfeeding, where you could find out important information, and also went to courses in Poland. I got to one of the lectures where there was just breastfeeding and childcare. Although I had experience with the first child, at this lecture it turned out that I even did something wrong with the first child while breastfeeding was new for me. In Ukraine, they didn’t tell me that—they didn’t tell me how to feed. I didn’t know how to do that. My breasts were just full of milk, they told me to express breastmilk, and at this lecture, I was told that it is necessary to give one breast and the other breast to the child to feed. I didn’t know that; I always gave one and for the second feeding to another one, that’s how it was (Refugee, child was born in Poland after the war started).*

A small number of refugees also received family support for cooking and feeding their infants, nutrition information, counseling by lactation counselors’ breastmilk from the milk bank, and psychological and mental health support from friends and organizations. A few refugees also said they received CMF donations.

### 3.10 Suggestions from participants on infant feeding support that is needed

When mothers were asked what they needed to help them feed their child during the war, breastfeeding information and support were the most frequently reported for both refugees and participants in Ukraine. Mothers said that breastfeeding information and support can be provided through lectures, courses, consultations (either face-to-face or online), social media, and hotlines. Specifically, they said that pregnant women and mothers of newborn infants need information and support specific to issues surrounding early initiation of breastfeeding, the importance of breastfeeding, when breastmilk comes in after giving birth, how to breastfeed, special diets for breastfeeding mothers, and how to overcome breastfeeding challenges such as how to increase breastmilk supply when the mother is stressed. Mothers also said that healthcare workers in maternity hospitals in Ukraine should be trained on the importance of breastfeeding and how to provide breastfeeding support to mothers. In addition, participants said that it was necessary to have lactation consultants in maternity ward in Ukraine to provide breastfeeding support to mothers who have just given birth and to address problems they face when starting out to breastfeed, as this participant in Ukraine reported:

*It’d be great to have at least one consultant, probably at the maternity hospital, to help me find the right positioning for breastfeeding. For people to be able to come and get help if they need it. Many give up on breastfeeding because they can’t find the right positioning. They can’t do it, so they decide their milk supply is insufficient. Studies have shown that there are few women who are unable to nurse a child for medical reasons. It’s mostly because they failed to find the right positioning, the infant couldn’t latch on their breasts… I mean, I’d like Ukraine to implement a government program, allocate the budget, and give a salary to this person who could help in the maternity hospital (Participant in Ukraine, child was born in Ukraine before the war).*

For those in Poland, women said they needed breastfeeding support, especially just after birth, in Ukrainian or Russian language, as this refugee mother explained:

*If we are talking about the topic of breastfeeding, it is support for pregnant women or those who have already given birth so that this process is established immediately and correctly. If the hospital declares that it provides support to people from Ukraine who fled the war, then it would be very good if there was a person in the hospital who could help with the issues of breastfeeding. When I was lying in the ward, a Polish midwife came to the Polish woman and helped her with the infant, and I heard it. I felt sad because no one came to me. I did not have time and did not know where to find a Ukrainian- or Russian-speaking specialist who could help me in a familiar language. And I was very sad that no one could help (Refugee, child was born in Poland after the war started).*

Refugees and participants in Ukraine said that they believed that providing CMF was needed in some circumstances as some mothers do not have enough breastmilk supply and that CMF is expensive. Women also said that they needed support in the form of complementary food for their children, such as porridge, puree, fresh fruits, and readymade meals for infants over 6 months.

Many participants in Ukraine and some refugees reported a need for psychological support. Participants said that because of the war, both mothers and their children were feeling anxious and trying to cope with the new realities of war. They felt emotionally unstable, were afraid of the explosions and shelling (for those in Ukraine), and needed psychological support, as this participant illustrated:

*We encountered a lot of people, we really want to help everyone, to say at least a kind word. But people are so closed in themselves that they don’t even want to listen to anything. All this requires time and some kind of support. Perhaps people want support from their loved ones, but they are not there, or they are far away. And there should probably be a special approach from a stranger because human injuries are very complex. I can’t even find the words to express what they need. Maybe just sit, hug, and not even say anything. I think they need such help. They definitely need psychologists, that’s 100% (Refugee, child was born in Poland after the war started).*

Only participants in Ukraine reported needing feeding support, specifically information on complementary feeding and what they should be feeding their child. They also wanted a platform for mothers who could not breastfeed to contact breastfeeding mothers who are willing to breastfeed the infants of other women (wet nurses). They also said that husbands and grandmothers should be given information on breastfeeding so that they can effectively support breastfeeding mothers. A couple of mothers also spoke about the need to improve access to high quality complementary food and fresh vegetables for their children. Providing shelters in each neighborhood suitable for families with infants was also a need that some participants in Ukraine mentioned. They wanted a shelter where people can stay during an air raid alarm, with a comfortable space for mothers to breastfeed and prepare their children’s food, as this participant in Ukraine reports:

*It would be nice if there were bomb shelters equipped so that women with children could go down there without worrying whether they’d be able to feed them there. A place for feeding and changing clothes, with napkins, diapers, and CMF, so that moms could mix their infant’s CMF, feed the infant, wipe it, change its diapers, something like that. I had to cover myself and feed the infant in front of everyone because there was nowhere to go. There is one room for everyone (Participant in Ukraine, child was born in Ukraine after the war started).*

Refugees in Poland also recommended creating a center for assistance for people in emergency situations in Poland, specifically to assist pregnant women, mothers, and women who are there without their male partners and relatives. This center would provide information on where people can go for help, including where to go see a doctor, and should be staffed by Ukrainian and/or Russian speakers:

*It would be good if these mothers were provided with informational assistance, help from psychologists, and advice on what to do with their children. For example, I don’t know where to call if my child has a fever. I can’t explain what I need at the pharmacy… In this way, it is difficult (Refugee, child was born in Poland after the war).*

## 4 Discussion

This qualitative study with 75 Ukrainian women during the first year of the conflict is one of very few documentations of how the war in Ukraine has affected new mothers’ experiences of feeding their infants. It provides insights into the type and extent of support that is offered to participants in the Ukrainian war zone and in Poland. In addition, it captured insights on additional assistance mothers believe are needed for women to be able to feed their infants as recommended. Their experience highlights that the humanitarian response has fallen short of meeting the known and urgent needs of infants and their mothers at a time of extreme vulnerability. Their experiences also show that humanitarian agencies have not been well prepared to apply learning’s from previous emergencies, including modifying humanitarian responses from conflicts in low- or middle-income countries for high-income country settings.

The significance of our study lies in the fact that is the only study so far that has highlighted the IYCF needs of women and their infants affected by the Ukraine war. The study provides information that the donor community and the humanitarian agencies can use to inform how they are supporting the emergency in Ukraine and in similar contexts. The study highlights the following key findings.

First, many of the women interviewed confirmed that their infant feeding practices were disrupted by the conflict, and that fear, anxiety, and stress were among the main causes. Many mothers believed that stress from the war would affect their milk supply. This is in line with recent evidence that suggests how perceived insufficient milk supply is the most common problem in breastfeeding and is the main reason for women to stop breastfeeding early (24, 25). While stress does not reduce milk production, it can result in a slowing of the let-down and flow of milk (26), meaning that infants may take longer to feed, want to feed more frequently, and are fussier when feeding. This together with behavior changes common in emergencies such as wanting to be held more and waking more overnight is often behind women’s concerns about their milk supply (27). This reaffirms the importance of providing breastfeeding counseling and support so that women can understand their infant’s behavior changes, regain confidence in their ability to breastfeed and continue breastfeeding (25, 28, 29).

Second, many of the pregnant women who gave birth after the war started and had planned before the war to exclusively breastfeed reported they had to change their plans. Instead of breastfeeding exclusively, they were now supplementing breastmilk with CMF. Some women never initiated breastfeeding, even though they had planned to breastfeed before the war. Participants attributed this in part to the lack of breastfeeding support from the health professionals in birth clinics. For the respondents in Poland, language was an additional barrier to effective breastfeeding support in birth clinics, highlighting the additional challenges that refugee women face in accessing lactation support and benefiting from it when available. These findings are in line with previously published evidence where access to skilled and knowledgeable health professionals has been lacking in emergencies and other contexts, and the lack of support for establishing breastfeeding created greater vulnerability among this already highly traumatized group (30–34).

Third, some participants (in Poland and Ukraine) had concerns about their ability to continue purchasing CMF due to increasing costs and the difficulty of preparing it in a safe way. At the same time, widespread and sometimes unnecessary donations of CMF were cited as the principal infant feeding support received by many of the women living in Ukraine, including some breastfeeding mothers who said that they were forced to receive it. This is in contrast to what was reported by women in Poland where offer of CMF was unusual. This difference may be in part due to very high number of local and international humanitarian agencies providing aid in Ukraine directly to the populations affected by the active conflict, in contrast to the situation in Poland where aid was delivered via the government in selected locations only. While this study was not designed to document the health impact of CMF donations in disaster-affected populations, other studies have, for example, the uncontrolled distribution of CMF in 2006 after the Yogyakarta earthquake, resulted in a documented subsequent increases in diarrhea cases in a population that previously had high breastfeeding prevalence (35).

Indiscriminate donations were also noted in the 2015 European Refugee Crisis, where more than one million migrants and refugees who arrived in Europe received commercial complementary foods and other processed foods. Overall, mothers were dissatisfied with their infant feeding outcomes (36).

Fourth, only one-third of the mothers said that they received any information on infant feeding, and very few mothers received lactation support. Their experience shows that IYCF support provided by the various humanitarian actors and organizations during the current emergency is generally not in line with the standards of the OG-IFE (7). It highlights the need for agencies and organizations to adapt and improve their responses to new situations. In high-middle-income countries, it is important to ensure that the IYCF-E response includes, among others, lactation counseling services accessible across various contact points with mothers and their children, where suitable access to human milk banks in line with the OG-IFE recommendations (7) and a package of services for infants dependent on infant formula in line with the OG-IFE recommendations (7), provision of suitable complementary foods and/or resources (cash or voucher support) to access suitable complementary foods, safe spaces for safe breastfeeding and infant formula feeding preparation and feeding among others.

Fifth, many of the participants asked for breastfeeding counseling support, more information, and guidance on complementary feeding, as well as properly equipped and comfortable shelters to be able to feed their infants comfortably. This reinforces the findings of a 2014 review by Action Contre la Faim (ACF) which identified the importance of “infant-friendly spaces” in emergency evacuation centers (37). “Infant-friendly spaces” were shown to facilitate a comprehensive approach to supporting pregnant and lactating women and young children in emergencies. They also served to prevent or reduce the negative effects of unsolicited and unmonitored distributions of breastmilk substitutes, as well as provide appropriate and sustainable solutions for non-breastfed infants.

Sixth, many participants in Ukraine and Poland talked about the need for psychological support as they were feeling anxious and stressed in dealing with the realities of the war. This finding is in line with other studies that suggest that conflict-affected populations suffer from psychological distress that directly impacts their ability to care for others, including infants and young children, hence the need for support and counseling (38, 39). Our study illustrates the challenges associated with the IYCF experiences of the women interviewed and how the initial IYCF-E response at the time of the study did not meet the needs of the beneficiaries. The findings are aligned with those of a recent review analyzing the challenges emergency responders still face in implementing IYCF-E programs and the barriers mothers face in breastfeeding or providing breastmilk in disasters, particularly in middle- and high-income countries (40, 41).

Several studies highlighted how maternal psychological distress affects women breastfeeding practices and outcomes (42–45) highlighting the importance of ensuring women affected by a conflict have access to qualified breastfeeding and psychosocial support as key to overcome their fears and concerns during conflicts and other emergencies.

A the same time evidence shows that breastfeeding support can help women to relactate especially when the infant is younger, the gap from when breastfeeding stopped and the woman wants to resume it is short, and the mother is strongly motivated to relactate (46). However, the success of relactation support can be challenged by the acute nature of the emergency and where prevalence of breastfeeding in a country is very low (46).

Various studies have shown that often the emergency response is hampered by indiscriminate donations of CMF, women do not receive adequate support, and are not provided with safe spaces to breastfeed their infants. In studies that reviewed emergencies (35, 36, 40), infant feeding support services, when available, did not meet the global standards or recommendations (40, 41).

Our findings are subject to several limitations. First, most interviews were conducted by phone or on web-based platforms, while some were conducted face-to-face. This lack of consistency in interviewing modalities might have introduced bias such as lack of visual cues in interviews conducted by phone or on web-based platform, or that participants could have felt more comfortable and relaxed by one interview method vs. the other (47, 48). Second, although we tried to recruit as diverse a sample as possible within Ukraine and Poland, our sample was heavily skewed toward highly educated urban mothers. Evidence from low- and middle-income countries has shown that women with no educational attainments have a high level of breastfeeding practices compared with other women with any category of education (49). At the same time, the use of CMF is generally higher among women with higher education levels across in these contexts (49). In Ukraine, however, mothers’ higher education seems to have positive rather than negative effects on breastfeeding practices. A previous IYCF study among those displaced during the conflict in 2015 (15) reported that mothers’ completed higher education was associated with significantly increased the odds of ever breastfeeding and decreased the odds of bottle feeding. Third, we chose to use a two-step rapid analysis approach using a structured template with Microsoft Excel as it takes less time than traditional thematic analysis (21). Although this rapid analysis approach may not provide a deeper understanding compared to traditional thematic analysis, studies have shown that the rapid analysis approach is still methodologically rigorous (22). Fourth, we found that some interviews were shorter than others. The shorter interviews were conducted by one specific interviewer who did not ask study participants for clarification or elaboration, resulting in less detailed, poorer data quality for those interviews.

The IYCF-E response interventions and resources serving women and their babies affected by the war in Ukraine at the time of the study largely failed to achieve its basic objectives. At the same time, no IYCF-E interventions and investments were mentioned or documented in Poland at the time of the study. This is despite (i) globally endorsed guidance on how to provide IYCF-E support, (ii) World Health Assembly resolutions stating that proper planning must be in place to provide support, (iii) experience from past emergencies in Ukraine showing that support is needed (15), and (iv) an emergency response with a plethora of resources and involving neighboring European middle- and high-income countries. This study suggests that more implementation research is needed to understand and identify why empirical evidence highlights this recurrent gap in emergency responses.

These findings provide evidence of the need for the global community, including donors, governments, UN agencies, and international and national agencies, to rapidly address the situation by ensuring that mothers and their infants affected by an emergency such as that in Ukraine receive comprehensive, appropriate, and timely IYCF support with an IYCF-E response plan that is context specific and responds to the infant feeding challenges of the Ukrainian women. The study demonstrates the urgent need to scale up the IYCF-E response to reach all women and their infants to meet their nutritional needs. For example, there is a need to engage and invest in local networks of lactation counselors, national organizations, and health professional organizations across Ukraine to increase the reach and coverage of IYCF services and especially strengthen lactation support in birth clinics and hospitals. Regular assessments of IYCF practices and needs of women and their infants affected by the conflict through both quantitative and qualitative research methods are needed to monitor effectiveness of these efforts. Finally, further investigations including larger representative quantitative studies would be useful where it is safe and there is access, to estimate the prevalence of key IYCF-E indicators and practices to establish the quantitative baseline from which success or failure can be measured.

## 5 Conclusion

In all settings, emergencies shift infants and young children and their caregivers into a “high risk” category by increasing the risk that breastfeeding will be reduced or ceased and that there will be limited or no access to a timely, safe, affordable, and appropriate diet for the child. Planning and preparing for IYCF-E support is essential for all country settings, and needs integration into mainstream responses everywhere, yet as shown above, this is deficient in the Ukraine response. While promoting and supporting breastfeeding and complementary feeding practices, it is crucial that CMF supplies are provided judiciously, and their distribution carefully coordinated and targeted to those with specific needs.

In Ukraine there is an immediate need to ensure that hospitals practice the WHO/UNICEF Baby- Friendly Ten Steps for Successful Breastfeeding and that lactation consultants are deployed to support women and their infants in health facilities, communities, and collective centers. At the same time, indiscriminate CMF donations and pressures on the mother not to breastfeed should not be tolerated. All government and non-government agencies working in Ukraine are encouraged to make IYCF materials on breastfeeding and complementary feeding widely available to women and families and help them access the lactation consultant hotline. It is essential to make locally accepted complementary foods (and CMF when needed) accessible and affordable during the war and displacement. For refugees, it is crucial that interpretation services are available at the hospitals and other relevant health facilities to remove language barriers related to essential health and nutrition services and support.

## Data availability statement

The raw data supporting the conclusions of this article will be made available by the authors, without undue reservation.

## Author contributions

AI, TS, and MO’B contributed to the concept and the design of the study. OB contributed to structuring the manuscript and reviewed all the sections of the manuscript. AI and CW performed the data processing, analysis, and drafted all the sections of the manuscript. JS, MV, KG, BW, and AW reviewed all the sections of the manuscript. BW and AW contributed to the design of the study tools. All authors contributed to manuscript review and approved the final version.
